# The role of keratins in modulating carcinogenesis via communication with cells of the immune system

**DOI:** 10.15698/cst2019.04.184

**Published:** 2019-03-23

**Authors:** Inês Sequeira, Fiona M. Watt

**Affiliations:** 1 Centre for Stem Cells & Regenerative Medicine, King's College London, Guy's Hospital, Great Maze Pond, London SE1 9RT, UK.

**Keywords:** cancer, keratin, Krt76, regulatory T cells, immunoregulation, anti-tumour

## Abstract

Keratins are intermediate filament proteins expressed by epithelial cells and provide mechanical support for diverse epithelia. In our recent study (Sequeira *et al.,* Nat Comm 9(1):3437), we analysed the role of keratin 76 (Krt76) in inflammation and cancer. Krt76 is expressed throughout embryonic development in the differentiated epithelial layers of a subset of stratified epithelia including tongue, palate and stomach. It is significantly downregulated in human oral squamous cell carcinoma (OSCC), correlating strongly with poor prognosis. We have shown that Krt76^−/−^ mice exhibit systemic inflammation with increased levels of circulating B cells, regulatory T cells and effector T cells. When mice are given a chemical carcinogen in the drinking water, tongue and gastric cancer formation is accelerated in Krt76^−/−^ mutant mice. Our data suggest that the increased tumour susceptibility of Krt76^−/−^ mice is in part due to the enhanced accumulation of regulatory T cells in the tumour microenvironment. Our results support the notion that keratins, in addition to their function as cytoskeletal components, regulate immunity and affect tumour susceptibility of epithelial cells.

The connection between inflammation and cancer has been well established; however, little is known about the underlying mechanisms in the context of keratin loss. Keratins belong to a large family of intermediate filament-forming proteins with a crucial role in maintaining epithelial integrity. Keratins are also involved in additional cellular functions, including apico-basal polarisation, regulation of intracellular signalling pathways, cellular motility, cell size control, cell proliferation, and intracellular transport. Therefore, defects in keratins can lead to loss of cell integrity and are linked to a variety of epithelial disorders and cancers. In addition, there is growing evidence that keratins can act as regulators of inflammation and immunity in multilayered epithelia.

Using Krt76-deficient mice (Krt76^−/−^), we have demonstrated an interesting and novel role for Krt76 in keeping the immune system in check and preventing oral and gastric cancer. In our study (Sequeira *et al.*, Nat Comm 9(1):3437), we have shown that upon loss of Krt76, mice develop a systemic inflammation, characterised by spleen and lymph node enlargement, increased numbers of B cells, of CD4^+^ CD44^high^ CD62L^low^ effector T cells (Teff) and of CD4^+^ CD25^+^ Foxp3^+^ regulatory T cells (Tregs), together with an increase in circulating pro-inflammatory cytokine levels. Several cytokines, including IL-6, IL-10, and TNFα, were found at increased levels in the serum, tongue, and squamous stomach of Krt76^−/−^ mice, suggesting that the loss of Krt76 leads to increased inflammation both locally and systemically.

To discover whether the functionality of Tregs and Teff differed in Krt76^−/−^ and control mice we performed *in vitro* cell suppression assays. Functional and phenotypical analysis of the T cell populations showed that not only were the numbers of Tregs increased in Krt76^−/−^ mice, but they also inhibited Teff proliferation more effectively than control Tregs (**Figure 1**). The increased suppressive capacity of Tregs from Krt76^−/−^ mice correlated with increased capacity to inhibit pro-inflammatory cytokine production and increased expression of the Treg functional suppressive markers CD39 and CD73. In addition, the proliferative activity of Teff from Krt76^−/−^ mice was lower than that of control Teff. CD39^+^ Tregs are known to suppress T cell proliferation and inflammatory cytokine production more efficiently than CD39^−^ Tregs.

**Figure 1:**
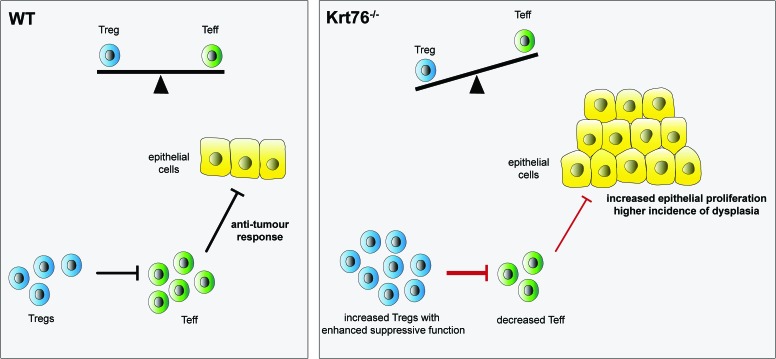
FIGURE 1: Summary schema of the balance between regulatory T cells (Treg) and effector T cells (Teff). Increased numbers of Tregs and their increased suppressive function in Krt76^−/−^ mice leads to a decreased antitumour immune response.

Recent advances in the field support the view that Tregs play a major role in the tumour microenvironment and contribute to tumour growth by controlling the antitumor immune response (**Figure 1**). We therefore tested the highly suppressive role of Tregs from Krt76^−/−^ mice in carcinogenesis. Krt76^−/−^ mice do not develop spontaneous tumours. We administered a synthetic carcinogen, 4-nitroquinoline *N*-oxide (4NQO), in the drinking water for 16 weeks and monitored tumour incidence for a total of 28 weeks. 4NQO treatment led to hyperplasia in the oral epithelium of control mice from 8 weeks of treatment, followed by dysplasia from 10 to 12 weeks, and the development of oral squamous cell carcinoma by 22 weeks. Krt76, known to be downregulated in human OSCC, was also found downregulated in the lesions. However, Krt76^−/−^ mice developed oral and squamous stomach tumours at an increased frequency relative to wild-type mice. Therefore, genetic ablation of Krt76 protein increases tumour incidence in tongue and squamous stomach.

No histological changes or epithelial barrier defects were observed in Krt76^−/−^ tongue or stomach epithelia, despite the fact that loss of Krt76 in the skin leads to impairment of the epithelial barrier. Therefore, the increase in tumour incidence is not due to increased carcinogen penetration. Instead, the increased susceptibility to developing cancer of the oral cavity and squamous stomach was correlated with an exacerbated inflammatory response to the carcinogen. We found that 4NQO-treated Krt76^−/−^ mice exhibited higher increases in serum levels of IFNγ, IL-4, IL-6, IL-10, and TNFα than control mice. Previous studies have shown that a large number of Tregs are present in tumours and draining lymph nodes. Analysis of the tumour microenvironment within hyperplastic and dysplastic lesions showed increased immune cell infiltrate, higher numbers of infiltrating Tregs and decreased ratios of Teff, suggesting a higher inhibition of Teff in the Krt76^−/−^ tumours. Furthermore, carcinogenesis could be further increased in Krt76^−/−^ mice when the number of Tregs was elevated experimentally using DEREG chimeric mice. This supports the concept that the increase in Tregs is correlated with increase of tumour incidence. We also obtained evidence that in human OSCC, increased Foxp3^+^ Tregs are found in Krt76-deficient regions.

We therefore suggest that upon loss of Krt76 and the consequent increase in Tregs, there is a failure in anti-tumour immunity that may be caused by an exaggerated suppression of anti-tumour-associated antigen-reactive lymphocytes. This is mediated by Tregs that dampen the anti-tumour response of activated T-cells, leading to an inhibition of the anti-tumour response (**Figure 1**).

## CONCLUSION

According to our findings, Krt76 is not only a cytoskeleton protein but also regulates immunity in mice. Krt76^−/−^ mice exhibit a marked inflammatory phenotype with systemic components and are more prone to tumourigenesis, suggesting a previously unidentified role for Krt76 in regulating immunity and emphasising its importance in tumour progression.

The response of Krt76^−/−^ mice to carcinogen included an exacerbated induction of inflammatory cytokines and enhanced accumulation of Tregs in the tumour microenvironment, leading to a drop in anti-tumour response correlated with an exaggerated suppression of the anti-tumour-associated antigen-reactive and increased epithelial cell proliferation (**Figure 1**, Krt76^−/−^).

The inability to resolve chronic inflammation is considered one of the triggers of carcinogenesis, and a major obstacle to successful tumour immunotherapy. Various strategies have been applied towards therapeutic modulation of Treg function by depleting Tregs, by attenuating Treg suppressive function, or by rendering Teff refractory to Treg-suppression. In this regard, our study highlights the importance of keratins as immunomodulators and the potential significance of Tregs in carcinogenesis, making them potentially attractive targets for new cancer therapies linked to chronic inflammation.

Future studies will give us a better understanding of the mechanism by which loss of Krt76 regulates carcinogenesis by modulating the immune system and help decipher the newly defined role of keratins in immunoregulation.

